# P10 Psychologist? But the pain is not all in my head…

**DOI:** 10.1093/rap/rkac067.010

**Published:** 2022-09-28

**Authors:** Alex Orchard

**Affiliations:** Evelina London Children's Hospital, Guy's and St Thomas NHS Foundation Trust, London, United Kingdom

## Abstract

**Introduction/Background:**

Chronic pain has been dubbed a place where mental and physical health meet. The emotional and social needs of young people experiencing Chronic Pain can be vast, yet families often fall through cracks in services. Other families receive a plethora of support options from psychological practitioners, allied health professionals and social care, but with laser focus on the medical answer and fix decline all potential referrals.

Historically psychology received numerous referrals for young people who declined to meet with us, leaving our colleagues and families feeling unheard, frustrated stuck and alone. How to support families/colleagues without seeing children individually?

**Description/Method:**

Ed (15-year-old male) experiencing paralysis and pain in dominant arm and hand:

• extensive investigations and specialist consultations sought, including scans and neurological opinion - nothing medically concerning identified.

• Previous referral to another tertiary psychology service made and group therapy offered.

• Over the course of the investigations reports of pain and A&E attendance increased, whilst school attendance decreased -> referred to chronic pain service.

Input offered at Evelina London:

• 2 x Multidisciplinary clinic (chronic pain diagnosis given at first appointment).

• 1 x Pain education workshop.

• Multiple physiotherapy and occupational therapy appointments.

• 3 x referrals to psychology over preceding 9 months with concerns about mood and engagement.

Progress:

Initial gains in first months

• Pain and paralysis reduced to dominant hand only.

• Strength and range of motion restored to shoulder and arm.

• School attendance increased.

However, in recent months A&E attendances and reports of pain were increasing, with service engagement decreasing.

Requests for individual psychology for Ed had been made by various team members, but Ed had not taken up offered psychology conversations or appointments. This contributed to expressions of frustration, worry and hopelessness in the team who wanted individual psychology for Ed “to move things forward”.

3rd Multidisciplinary Clinic appointment:

With all therapists and doctors in attendance the psychologist took time to explore Ed and his mother's daily lives outside pain, their interests, joys and skills. They explored the family's explanations for pain, experience of services so far, roles they felt investigations, doctors, occupational therapy, physiotherapy and psychology had, could and should play.

Amongst other things particular emphasis placed on:

• Privileging family's views.

• Using patient's language.

• Regular summarising.

• Placing professionals then family members in listening positions and exploring what each had heard the other say.

The psychologist endorsed Ed's view that psychology had no role to play in his care.

**Discussion/Results:**

Was Ed in desperate need of 1-1 psychology to move forward?

For this family the psychological intervention was with both family and healthcare system. The psychologist used a narrative approach, positioned as a curious (almost) "outsider", with the patient as an expert in their own care. Beginning with a foundation of identity outside pain, followed by facilitated witnessing of ideas and experiences for family and healthcare professionals. Thus, new insights, a shared understanding and plan could develop.

With this approach it emerged referrals to psychology coupled with no simple medical explanation and treatment fed fears professionals thought "pain was all in Ed's head". This shook confidence in the healthcare team and reinforced messages the family experienced family, school and friends as giving. Around this time Ed's progress stalled.

Whilst family described previous negative experiences of psychologists, current physiotherapy was described as developing "strength and mobility", occupational therapy as "really understanding" and giving "useful exercises/advice", cheerleading throughout a difficult journey. Doctors offered (another) detailed review of investigations and physical examination reiterating pain, but not damage were present. The psychologist thoroughly explored and all things psychology might add, none of which the family felt were useful. This choice was thoroughly validated and respected.

During the appointment "thinking break" (family not present) the team were surprised by the family's positive interpretations of care offered thus far. It was hypothesised not feeling believed/validated was a very difficult place to "recover" from and ideas generated of what validation/being believed would look like for this family. Given the family's vocalised block to progress was "the hand", perhaps is was a hand specialist rather than a psychologist?

The family were pleased to see joint occupational and hand therapy resulting in "full recovery". Throughout the professionals were offered regular psychological consultation and team discussion time.

**Key learning points/Conclusion:**

**There are many ways to peel the chronic pain orange.**

When faced with often overwhelming needs of young people living with chronic pain and sometimes overwhelming needs of referring health and social care systems it can be hard to hold onto this idea. Initially referrers thought individual psychology was indicated, what was offered and created change was a psychologically informed multidisciplinary appointment in which the family's worries were given space, validated and answered, whilst their right to decline psychological support was validated and bolstered. Alongside ongoing psychological consultation to professionals.

**Pain is all in my head, no-one believes me, no-one understands.**

Are powerful ideas that can be detrimental to family-healthcare relationships, whether stated directly, implied or internally voiced. They can represent a significant barrier to families accessing psychology directly.

We may wholeheartedly believe people experience pain that cannot reliably be seen or measured externally, but that does not mean we are experienced that way. For me this case highlighted the importance of

• checking out beliefs about symptoms.

• gauging whether ongoing/regular validation and reassurance is needed and in what form.

• even celebrating someone's successes in the face of pain even suggesting someone can move on with their life can be experienced as invalidating/not getting it. Families need a voice in their won paths forward.

**Is what you thought you said what they thought you meant?**

Whether with families or professional you can never underestimate the value of checking whether the same thing was heard differently.

This is written from the position of a psychologist embedded within a predominantly non-pharmacological multidisciplinary chronic pain service, which informed the intervention. Other cases have invited/necessitated different approaches. It would be interesting and helpful to invite discussion around the variety of ways similar/different road blocks have been bypassed, removed or otherwise.

